# Oxidative protein folding in the intermembrane space of human mitochondria

**DOI:** 10.1002/2211-5463.13839

**Published:** 2024-06-12

**Authors:** Christine Zarges, Jan Riemer

**Affiliations:** ^1^ Institute for Biochemistry University of Cologne Germany; ^2^ Cologne Excellence Cluster on Cellular Stress Responses in Aging‐Associated Diseases (CECAD) University of Cologne Germany

**Keywords:** ALR, IMS, MIA40, mitochondria, oxidative protein folding, protein import

## Abstract

The mitochondrial intermembrane space hosts a machinery for oxidative protein folding, the mitochondrial disulfide relay. This machinery imports a large number of soluble proteins into the compartment, where they are retained through oxidative folding. Additionally, the disulfide relay enhances the stability of many proteins by forming disulfide bonds. In this review, we describe the mitochondrial disulfide relay in human cells, its components, and their coordinated collaboration in mechanistic detail. We also discuss the human pathologies associated with defects in this machinery and its protein substrates, providing a comprehensive overview of its biological importance and implications for health.

## Graphical abstract

Mitochondria possess different pathways to import proteins into their subcompartments. Import into the intermembrane space is, for many proteins, driven by the mitochondrial disulfide relay, a machinery that employs disulfide formation and oxidative protein folding as driving force. In this review, we introduce this machinery in human cells and highlight novel recent findings.

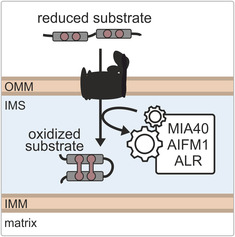

**Abbreviations**
ABCB8: ATP binding cassette subfamily B member 8
AIFM1: apoptosis‐inducing factor 1
ALR: augmenter of liver regeneration
APE1: AP‐endonuclease 1
ATP: adenosine tri phosphate
CCS1: copper chaperone for SOD1
CHCHD: coiled‐coil‐helix‐coiled‐coil‐helix domain containing
Cox12: cytochrome *c* oxidase subunit
DNA: deoxyribonucleic acid
DPP8/9: dipeptidyl peptidase 8/9
FAD: flavin adenine dinukleotide
GLRX1: glutathione reductase
GRX1: glutaredoxin 1
H_2_O_2_
: hydrogen peroxide
H*c*S: holocytochrome *c* synthase
HSP: heat shock protein
IMM: inner mitochondrial membrane
IMS: intermembrane space
ITS: IMS targeting signal sequences
kDa: kilo Dalton
MIA40: mitochondrial import and assembly 40 kDa
MICOS: mitochondrial contact site and organizing system
MICU1/2: mitochondrial calcium uptake 1/2
MISS: mitochondrial IMS sorting sequence
MTS: mitochondrial targeting sequence
mV: milli volt
NAC: nascent polypeptide‐associated complex
NAD(P)H: nicotinamide adenine dinucleotide (phosphate) hydrogen
NDUFB7: NADH dehydrogenase [ubiquinone]1 beta subcomplex subunit 7
OMM: outer mitochondrial membrane
RAC: ribosome associated complex
RNC: ribosome nascent chain
ROS: reactive oxygen species
SOD1: superoxide dismutase 1
TIM: translocase of the inner membrane
TMD: transmembrane domain
TOM: translocase of the outer membrane
TXN1: thioredoxin 1
Ubc4/5: ubiquitin‐conjugating enzyme E2 4/16 kDa
UPRnm: unfolded protein response activated by mistargeting of proteins
UPS: ubiquitin‐proteasome system

## The mitochondrial intermembrane space

Mitochondria provide the majority of cellular ATP, contribute to the synthesis (e.g., porphyrins, steroid hormones, and iron–sulfur clusters) and turnover (e.g., amino acids, fatty acids) of biomolecules and impact on cellular ion homeostasis (e.g., iron, copper, and calcium ions) [1]. To fulfill their functions, mitochondria are tightly embedded into cellular signaling networks [2]. They release reactive oxygen species and metabolites for signaling and modulation of cell fate decisions, mitochondrial DNA to initiate immune responses, and proapoptotic factors to drive apoptosis. Mitochondrial function depends on their more than 1500 different proteins that are distributed throughout its four subcompartments, the aqueous matrix and intermembrane space (IMS), and the mitochondrial inner (IMM), and outer (OMM) membranes [3, 4, 5].

The IMS is embedded between the IMM and OMM and subdivided into the peripheral IMS, which faces the OMM, and the cristae space [6]. Cristae junctions separate both subcompartments and might provide a diffusion barrier for proteins, which would result in two subcompartments with a distinct protein and thus functional repertoire. The IMS contains about 150 soluble proteins and many proteins that are embedded in the IMM and OMM but expose their functional domains toward the IMS [3, 4, 7, 8, 9]. Proteins of the IMS fulfill functions in protein import and quality control, respiratory chain assembly and maintenance, ion transport, signaling and the initiation of cell death, transport and metabolic turnover of small molecules including phospholipids and heme precursors and controlling mitochondrial morphology and dynamics [1, 5].

## Protein import into the IMS

**Fig. 1:**
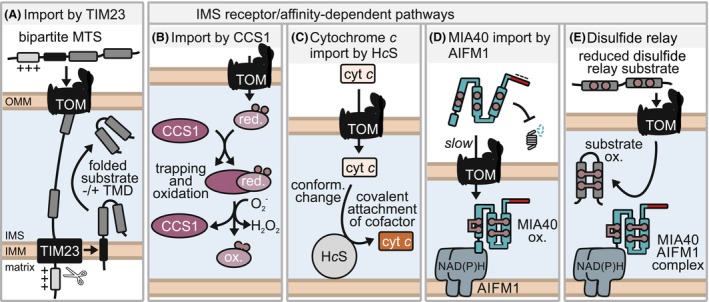
Protein import into the IMS. (A) Proteins with a bipartite MTS engage TIM23 and are laterally released into the IMM. Subsequently, the MTS is cleaved off and the substrate remains anchored in the IMM via a transmembrane domain (TMD). Alternatively, further processing steps can lead to the release of the substrate to the IMS. (B) Mature CCS1 in the IMS interacts with immature CCS1‐ or SOD1‐precursors. This leads to folding and disulfide bond formation in an ROS‐dependent manner. (C) Noncovalent interaction with holocytochrome *c* synthase (H*c*S) enables cytochrome *c* folding, heme cofactor incorporation and import. (D) MIA40 is imported into the IMS via AIFM1. A C‐terminal negatively charged stretch stabilizes MIA40 during the slow import process in the cytosol, while an N‐terminal stretch allows interaction with the AIFM1 dimer in the IMS. The AIFM1 dimer only forms in the presence of NAD(P)H. (E) Substrates of the disulfide relay pathway require interaction with the MIA40‐AIFM1 complex. They enter the IMS in a reduced state and are concomitantly to import oxidatively folded and thereby trapped in the IMS.

Almost all mitochondrial proteins are synthesized at cytosolic ribosomes and guided to the translocase of the outer membrane (TOM) by only poorly understood processes. From there, dedicated protein import pathways shuttle the precursors to the mitochondrial subcompartments [10]. For import into the IMS, precursors mainly rely on either of two routes: the TIM23‐dependent pathway or a diverse set of IMS receptor/affinity‐dependent pathways (Fig. 1).

To engage the TIM23 pathway (Fig. 1A), substrates contain an N‐terminal bipartite mitochondrial targeting sequence (MTS) that consists of a partially positively charged amphipathic helix followed by an alpha‐helical transmembrane segment [10]. Upon import, the TIM23 translocase in the IMM is engaged resulting in a lateral release and anchoring of substrates in the IMM using this transmembrane segment [11]. Parts or the entire bipartite MTS can be processed and thereby removed resulting in mature proteins that are embedded in the IMM facing the IMS or are soluble IMS proteins, respectively [12, 13].

Intermembrane space receptor/affinity‐dependent pathways employ a diverse set of import receptors inside the IMS (Fig. 1B–E). Substrates of these pathways do not contain classical N‐terminal MTS but other features like conserved cysteines and hydrophobic patches that allow their interaction with dedicated receptor systems. Most substrates are small proteins and their import is comparatively slow.

For example, both human superoxide dismutase 1 (SOD1) and copper chaperone for SOD1 (CCS1), dually localized proteins between cytosol and IMS, rely on a CCS1‐dependent pathway for import (Fig. 1B) [14, 15, 16]. IMS‐localized and folded CCS1 provides an interaction and folding platform for incoming immature CCS1 and SOD1. Both proteins acquire a disulfide bond in the process, which is at least in part dependent on the local levels of reactive oxygen species (ROS). Consequently, increasing the levels of mitochondrial ROS also shifts the cellular distribution of both proteins and results in accumulation of increased levels of CCS1 and SOD1 in the IMS [14, 15]. There are indications that in the absence of CCS1, a small amount of SOD1 can also become imported by the mitochondrial disulfide relay (see below) [17] paralleling the situation in yeast where Ccs1 import depends on the disulfide relay [18, 19, 20].

Cytochrome *c* depends for its IMS import on the protein holocytochrome *c* synthase (H*c*S) (Fig. 1C) [21, 22, 23]. Cytochrome *c* enters the IMS in its unfolded state where it first interacts with H*c*S non‐covalently. For this import, still ill‐defined regions at the cytochrome *c* N‐ and C terminus are required. During cytochrome *c* folding, the heme cofactor is covalently attached with two thioether bonds to two specific cysteines in a CXXCH motif. This necessitates that those cysteines are maintained reduced throughout the import process. Deletion of H*c*S leads to accumulation of apo‐cytochrome *c* in the cytosol. Conversely, replacement of the heme‐binding cysteines still leads to a partial IMS import of (nonfunctional) cytochrome *c*, which can be increased upon H*c*S overexpression. Without insertion of the heme cofactor, cytochrome *c* remains instable and has a higher turnover than the mature form [24].

The protein MIA40 (mitochondrial import and assembly 40 kDa; in humans also CHCHD4) becomes imported through interaction with its partner apoptosis‐inducing factor 1 (AIFM1) (Fig. 1D) [25, 26]. To this end, human MIA40 carries an unstructured N‐terminal segment. This region interacts with AIFM1 but only if AIFM1 is present in its dimeric form. AIFM1 dimerization is induced by binding of NADH or NADPH [25, 27]. After import and folding, MIA40 remains in complex with AIFM1. In the absence of AIFM1, MIA40 can switch pathways and become imported by the mitochondrial disulfide relay, however, less efficiently (see below) [28, 29]. MIA40 import is very slow; some data indicate that its half import time might be as long as 90 min after synthesis [25, 27]. Consequently, an unfolded MIA40 precursor dwells for this time in the cytosol and has to be stabilized. The C terminus highly negatively charged region in MIA40 contributes to cytosolic stabilization via an unknown mechanism [26].

In addition, further proteins such as glutathione reductase (GLRX1), glutaredoxin 1 (GRX1), and thioredoxin 1 (TXN1) have been shown to be present in small amounts in the IMS [3, 4, 7, 8, 30]. The mechanisms driving their IMS import remain uncharacterized; however, they lack an MTS and will thus likely also rely on affinity‐dependent import pathways.

The most widely used affinity‐driven pathway is the disulfide relay (also MIA pathway) (Fig. 1E). To be targeted by the disulfide relay pathway, substrates rely on conserved cysteine pairs and the MIA40‐AIFM1 complex in the IMS. Upon import, the substrate cysteine pairs are oxidized by MIA40, which leads to the substrate retention and accumulation in the IMS. The remainder of this review will focus on the mitochondrial disulfide relay pathway.

## The mitochondrial disulfide relay

### The diverse landscape of human disulfide relay substrates

**Fig. 2:**
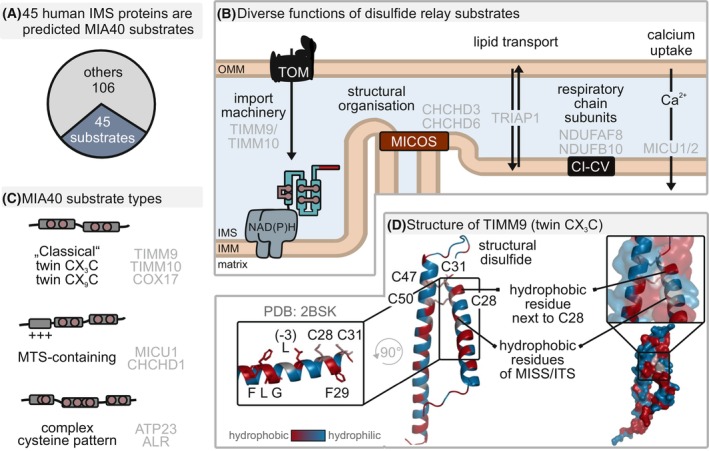
Diverse landscape of human disulfide relay substrates. (A) Around one‐third of human IMS proteins are predicted or have been demonstrated to be disulfide relay substrates. (B) Disulfide relay substrates are required for diverse functions in the IMS such as protein import, lipid transport, mitochondrial morphology and cristae organization, signaling, respiratory chain assembly and maintenance, or proteostasis. (C) “Classical” MIA40 substrates consist of a helix–loop–helix structure with conserved cysteines that are arranged in twin CX_3_C or CX_9_C motifs. Besides these classical substrates, there are MTS‐containing substrates or substrates with complex cysteine arrangements that have been discovered as disulfide relay substrates as well. (D) The structure of TIMM9 (PDB: 2BSK) displays features of a typical disulfide relay substrate with a twin CX_3_C motif. Hydrophobic residues (red) stabilize the helix–helix interface in addition to the structural disulfides.

Of the 150 soluble proteins found in the IMS, approximately 50 are substrates of the human disulfide relay pathway [3, 31, 32] (Fig. 2A). They fulfill diverse roles in respiratory chain assembly, as structural subunits of respiratory chain complexes, and the MICOS complex for the structural organization of the IMM, and in the transport of phospholipids (Fig. 2B) [3].

Most disulfide relay substrates are small in size (< 20 kDa) and consist of simple helix–loop–helix structures [33, 34, 35, 36]. Within these helices, conserved pairs of cysteine residues are positioned in twin CX_*n*_C motifs (most common, *n* = 3 or 9). The cysteines are placed in a way that in the mature protein, they face each other, and link the helices in two parallel disulfide bonds (Fig. 2C,D) [33, 37]. Together with hydrophobic residues, which mediate hydrophobic interactions to stabilize the helix–helix interface, the disulfide bonds provide enormous stability to the fold. The disulfide bonds are thereby partially covered and have a very negative redox potential that allows these proteins to persist in an oxidized state in the reducing IMS and even the cytosol [38, 39, 40]. This illustrates why it is important to tightly control the redox state of the substrate precursors *en route* to mitochondria and prevent their oxidation to avoid futile folding and accumulation of proteins in the cytosol.

Besides the abovementioned small disulfide relay substrates, a growing group of proteins emerged that do not form simple helix–loop–helix structures but as mature proteins contain more complex disulfide bond patterns (Fig. 2C). In some cases, these proteins even carry bipartite MTS. This group of proteins includes, for example, ATP23 [41], MICU1 and MICU2 [32], Rieske protein [42] as well as p53 [43], ABCB8 [44], and APE1 [45]. For some of these substrates, the precise mechanisms of disulfide formation and import are poorly defined.

Although the structural diversity of discovered disulfide relay substrates is constantly growing, a commonality of all substrates appears to be the positioning of at least one of their conserved cysteines in an α‐helix in close proximity to hydrophobic amino acids. This structural feature is important for their recognition by and interaction with the disulfide relay machinery (see below).

### Posttranslational import from the cytosol and its control by the proteasome and reducing systems

**Fig. 3:**
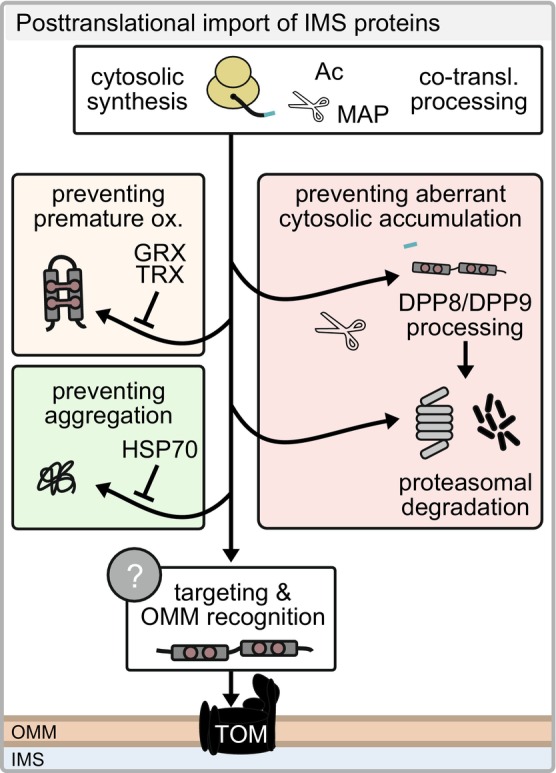
Quality control processes in the cytosol and posttranslational import into the IMS. Proteins are synthesized by cytosolic ribosomes and targeted to the mitochondria for import. A specific mitochondrial surface receptor for disulfide relay substrates is unknown so far. IMS precursors can be stabilized in the cytosol by chaperones, kept in an unfolded stage via the glutaredoxin and thioredoxin systems (GRX/TRX) as well as zinc binding, N‐terminally processed by DPP8 and 9, degraded in a proteasome‐dependent manner or accumulate and aggregate due to e.g. protein misfolding.

Most if not all disulfide relay substrates are posttranslationally imported (Fig. 3) [37, 46]. The synthesis of proteins at cytosolic ribosomes is supported by ribosome‐associated proteins that help to stabilize the nascent chain or mediate its co‐translational modification. This includes removal of the initiator methionine or addition of N‐terminal acetylation [47, 48, 49]. Both have also been reported for disulfide relay substrates [50, 51]. The stabilization of the emerging polypeptide chain is ensured by a network of ribosome‐associated chaperones [52]. These chaperones bind the emerging polypeptide chain to prevent misfolding. Ribosome‐associated chaperones include the nascent polypeptide‐associated complex (NAC), the ribosome‐associated complex (RAC), and HSP70 proteins [53, 54, 55, 56]. HSP70 chaperones have been shown to be important for the cytosolic stabilization of several different mitochondrial protein classes, such as MTS‐containing proteins, β‐barrel proteins of the OMM, and solute carriers of the IMM [57, 58, 59]. The disulfide relay substrate CHCHD3 has been shown to interact with HSP70 [60], and it is likely that also other disulfide relay substrates rely on the HSP70 machinery. While HSP70 and HSP90 show a broad substrate range, specificity for certain protein classes has been shown for the HSP40 co‐chaperones [58, 59, 61]. However, for disulfide relay substrates, no specific HSP40 co‐chaperone has been identified so far.

For disulfide relay substrates, the maintenance of a reduced redox state of cysteine residues in the cytosol is also important because disulfide formation or other modifications will prevent import of these substrates (Fig. 3). Cytosolic glutaredoxin (human GRX1) and thioredoxins (Trx1 and Trx2 in yeast) as well as the binding of zinc ions to the reduced thiols have been shown to be important factors in maintaining the proteins reduced [62, 63, 64].

The stabilization of mitochondrial precursor proteins is closely linked to their targeting and recognition on the mitochondrial surface. While no specific targeting factors for mitochondrial precursor proteins have been identified, HSP40/HSP70 chaperones bind to the receptor proteins TOM20, TOM22, and TOM70 and thereby mediate docking of precursor–chaperone complexes to the mitochondrial surface [57, 58, 59, 61]. Interestingly, disulfide relay substrates do not bind TOM receptor proteins but appear to interact directly with the pore‐forming subunit TOM40 [65, 66]. However, in yeast, they appear to be dependent on the TOM subunit Tom5 [66, 67]. An interesting case of mitochondrial targeting are the disulfide relay substrates MIC19 and NDUFB7. MIC19 contains a large N‐terminal so‐called DUF domain. Such a large domain would normally impair import by the disulfide relay; however, in the case of MIC19, N‐terminal myristoylation in the cytosol circumvents this [68]. Myristoylated MIC19 binds to TOM20, which aids in efficient import. NDUFB7 has been found to be myristoylated as mature protein inside mitochondria, and its recognition on the OMM might function similar to MIC19 [69].

In yeast cells, a large fraction of disulfide relay substrate precursors appears to be degraded by the UPS even prior to import into mitochondria [70, 71]. For Cox12, the E2 and E3 enzymes Ubc4 and Rsp5 have been shown to mediate its ubiquitylation and thus its proteasomal degradation [70]. Similarly, in human cells, disulfide relay substrate precursors are degraded continuously as well as upon impaired protein import [25, 27, 37]. This is reinforced by the N‐terminal processing of about one‐third of all human disulfide relay substrates by the cytosolic peptidases DPP8 and DPP9. After processing, they expose neo‐N terminals, which in most cases appear to be destabilizing [50]. This is thought to prevent any accumulation of these substrates in the cytosol and contribute to their exclusive IMS localization. However, the players involved in the recognition and degradation of human disulfide relay precursors remain unidentified.

### Beyond the TOM channel—encountering MIA40

**Fig. 4:**
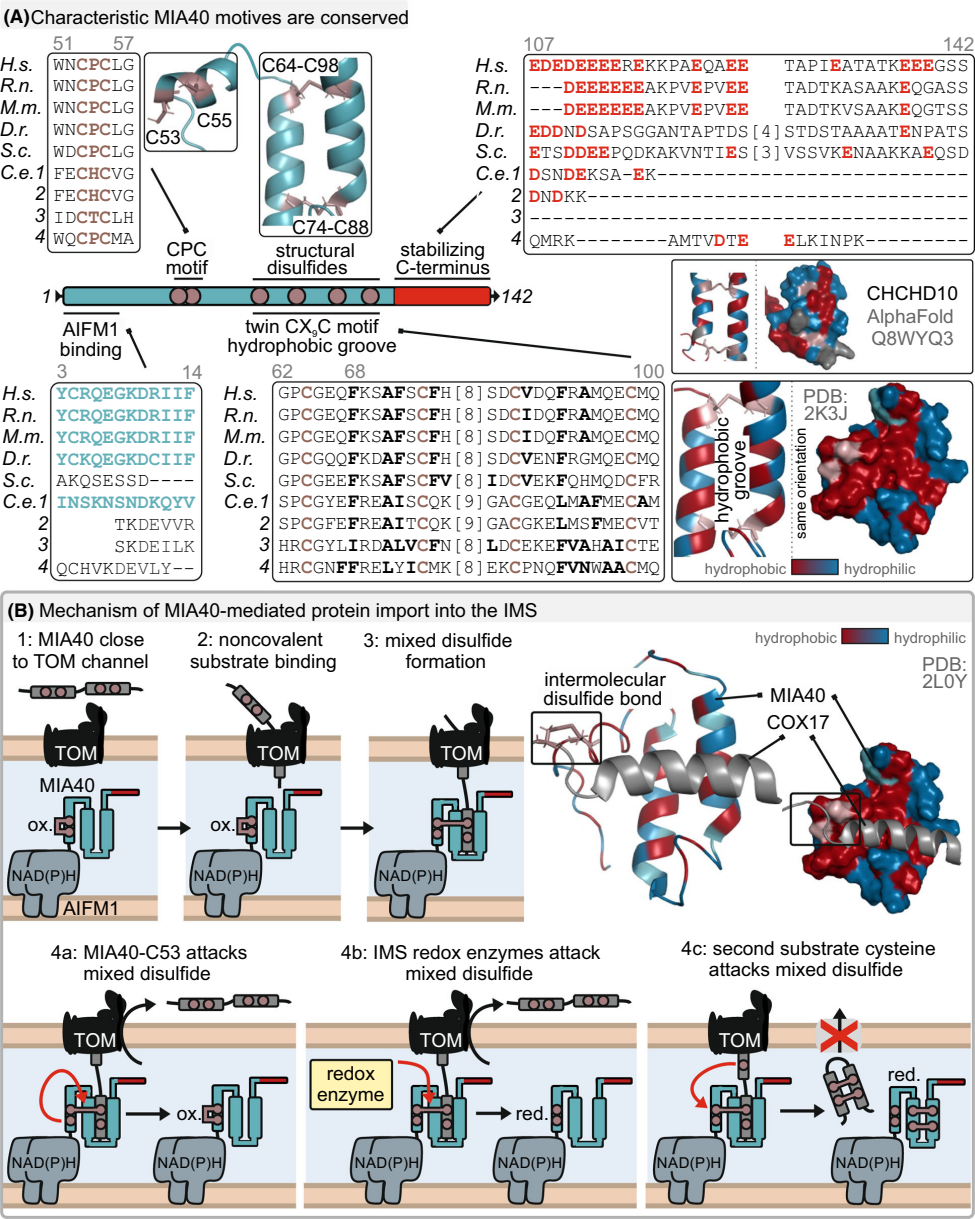
MIA40‐dependent oxidative protein folding. (A) Characteristic features of MIA40 are conserved. The redox‐active CPC motif, the compact core region with the structural disulfides and the hydrophobic groove, and the unstructured N‐terminus that is required for AIFM1 binding. The C‐terminus is negatively charged and important for MIA40 stabilization in the cytosol preventing its proteasomal degradation *en route* to mitochondria. The helix‐coil‐helix conformation exposes hydrophobic residues that are important for MIA40 chaperone function (PDB: 2K3J, red). (B) MIA40 is positioned adjacent to the TOM channel (1). Upon crossing the OMM, substrates interact non‐covalently with MIA40 (2). A specific substrate cysteine residue is positioned in close proximity to the CPC motif in MIA40, enabling its nucleophilic attack on the oxidized CPC motif. This leads to the formation of a mixed intermolecular disulfide bond (3, PDB: 2L0Y). The free cysteine residue 53 of MIA40 can attack the mixed disulfide resulting in release of reduced substrate and oxidized MIA40 (4a), the mixed disulfide can also be attacked by dedicated IMS redox enzymes (glutaredoxins, thioredoxins) resulting in reduced substrate and MIA40 (4b) or a second cysteine residue from the substrate enters the IMS through the TOM channel and attacks the mixed disulfide resulting in productive formation of a disulfide bond in the substrate and therefore preventing its backsliding into the cytosol (4c). The MIA40‐CPC motif remains reduced and needs to be reoxidized to be able to facilitate the import of a new disulfide relay substrate.

Upon passing through the TOM channel, disulfide relay substrates encounter the import receptor MIA40 (Fig. 4A). MIA40 is a small 142 amino acid protein with unstructured N‐ and C‐terminal regions, and a compact core that fulfills both a redox and a chaperone function [36, 37, 41, 72, 73, 74]. The unstructured N terminus serves in binding to AIFM1 during MIA40 import [25, 26]. Two isoforms of MIA40 exist that differ in the very N terminus but can both interact with AIFM1 [27]. The C‐terminal region is negatively charged and important to stabilize and protect MIA40 from proteasomal degradation in the cytosol [26]. The core of MIA40 consists of three helices, two are tightly packed in a helix‐coil‐helix conformation and connected by two disulfide bonds (MIA40 contains a twin‐CX_9_C motif) [36, 73]. This compact core exposes a patch of hydrophobic residues. By forming a shallow hydrophobic groove (F68, A71, and F72 in human MIA40), the functional unit of the holdase activity of MIA40 is formed [41, 73, 74, 75, 76]. A third short helix, positioned N‐terminally to the helix‐coil‐helix motif, is flexible and contains a redox‐active CPC motif (C53‐P54‐C55 in human MIA40). The CPC motif has a very negative redox potential of about 290 mV fitting well to the redox potentials of its substrates and the highly reducing redox milieu of the IMS [36, 38]. Importantly, the loss of both, the holdase as well as the redox activity affects MIA40 function, although the latter has more dramatic consequences in terms of substrate loss and cell viability.

Different lines of evidence suggest that MIA40 is positioned right behind the TOM channel (Fig. 4B.1). *In vitro* ribosome‐nascent chain (RNC) experiments predicted a minimal length of the nascent chain for MIA40 interaction that would precisely fit to an encounter of the first cysteine residue in the RNC with MIA40 right at the IMS‐side of the TOM pore [77]. The MICOS complex (a complex important for structuring the IMM, maintaining cristae junctions, and enabling IMM‐OMM contacts) has been proposed to serve in positioning MIA40 [78]. Likewise, it has been proposed that one function of the stable MIA40–AIFM1 complex that is formed during MIA40 import is the positioning of MIA40 close to the TOM channel [27]. This would point to a supporting role of AIFM1 in the disulfide relay, and indeed, the loss of AIFM1 can be compensated by overexpression of MIA40.

### Oxidation‐mediated protein import by MIA40

In the IMS, disulfide relay substrates first interact non‐covalently with MIA40. This interaction involves the hydrophobic groove in MIA40 and hydrophobic residues in the substrate (Fig. 4B.2/3). These residues are found directly next to a specific cysteine in the substrate, and at the −3/−4 and −7/−8 positions with respect to this cysteine, putting them on the same face of a helix. This motif of hydrophobic residues in the substrates have been termed MISS (mitochondrial IMS sorting sequence) or ITS (IMS targeting signal sequences) [79, 80]. Notably, in the mature folded protein, the motif is found at the helix–helix interface contributing to the stabilization of the substrate (Fig. 2D) [33]. Mutation of the MISS/ITS motif impairs import and folding of disulfide relay substrates [79, 80].

Through the hydrophobic interaction, the abovementioned substrate cysteine is positioned in a way that allows its nucleophilic attack on the redox‐active CPC motif of MIA40 [74, 81]. As a consequence of this attack, a covalent mixed disulfide linkage between the substrate and cysteine 55 in MIA40 is formed (Fig. 4B.3) [36, 81, 82]. In principle, three alternative steps can follow next: (a) The second cysteine (C53) in MIA40 can attack the mixed disulfide, releasing the substrate and MIA40 again in their reduced and oxidized states, respectively, (b) the mixed disulfide bond is attacked by a redox enzyme in the IMS such as thioredoxin or glutaredoxin, which reduces it and leaves both substrate and MIA40 behind in a reduced state, and (c) a second cysteine from the substrate attacks the mixed disulfide resulting in disulfide bond formation in the substrate leaving reduced MIA40 behind (Fig. 4B.4a–c) [36, 37, 74, 75, 76]. This last step leads to productive oxidative folding of the substrate. It relies, however, on the presence of a second cysteine in the substrate. The occurrence of this cysteine from the TOM channel during the vectorial import of substrates might take some time rendering the mixed disulfide bond a potential target for the reactions (a) and (b) described above [37]. The relative longevity of the MIA40‐substrate linkage compared with other systems for oxidative protein folding in the endoplasmic reticulum or the bacterial periplasm has in the past also aided the successful isolation and identification of substrates [32, 37, 76].

Many disulfide relay substrates possess multiple disulfide bond. It is likely that various MIA40 molecules work together on a single substrate, with one MIA40 possibly positioning the substrate while others facilitate the formation of additional disulfide bonds [81]. In particular for more complex disulfide bond patterns, the question arises whether MIA40 can serve as a disulfide isomerase. *In vitro* work supported such a function, in particular acting together with reduced glutathione albeit at a very low activity [75, 81, 83, 84].

Collectively, two factors contribute to disulfide relay‐dependent import: first, hydrophobic interactions that guide the substrate and MIA40 toward each other, and second the covalent interactions in a dynamic metastable complex between MIA40 and substrate, and the following disulfide formation. After disulfide formation, the oxidized and folded substrate is released and trapped inside the IMS (Fig. 4B.4c). The hydrophobic residues that are important to initiate the interaction with MIA40 are upon folding also buried. The CPC motif of MIA40 remains in a reduced state and needs to be reoxidized for a further round of oxidation‐dependent import.

### Keeping the electrons flow—reoxidizing MIA40

**Fig. 5:**
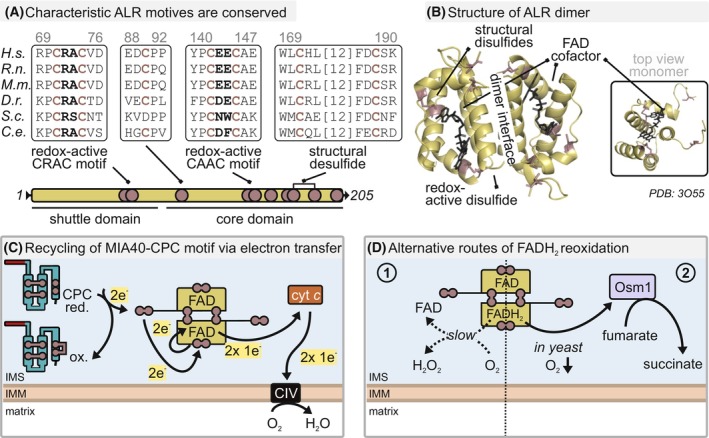
Reoxidation of MIA40 by ALR. (A) Characteristic features of ALR are conserved. The N‐terminal shuttle domain of ALR contains a redox active CRAC motif. The core domain forms a four‐helix bundle and binds a flavin cofactor noncovalently. It contains a redox‐active CAAC motif as well. (B) Two disulfide bonds and noncovalent hydrophobic interactions stabilize the ALR homodimer. The top view shows the four‐helix bundle and the loosely attached FAD. A fifth helix is fixed to the four‐helix bundle by a structural disulfide bond (PDB: 3O55). (C) The shuttle arm of ALR is important to bind into the hydrophobic groove of MIA40 which enables to place the oxidized CRAC domain close to the MIA40 CPC motif. C55 of MIA40 attacks the CRAC motif, resulting in a thiol‐disulfide exchange. Oxidized MIA40 and a reduced CRAC are the result. The shuttle arm interacts with the second ALR subunit and transfers its electrons to the redox active CAAC motif in its core domain in an inter‐subunit electron transfer reaction. The electrons are further transferred to the FAD cofactor. FADH_2_ is reoxidized by electron transfer to cytochrome *c*, which shuttles electrons subsequently to cytochrome *c* oxidase (CIV). (D) FADH_2_ can be reoxidized by molecular oxygen or in yeast by transfer to the metabolic electron acceptor fumarate reductase Osm1. These processes are much slower compared to the electron transfer onto cytochrome *c*.

MIA40 is reoxidized by the protein augmenter of liver regeneration, ALR (also human Erv1 or GFER) (Fig. 5A) [46, 81, 82, 85, 86]. ALR consists of two parts, an N‐terminal shuttle domain containing a CXXC‐active site motif (CRAC in human ALR), and a C‐terminal core domain [87, 88]. This core‐domain consists of a four‐helix bundle that holds a non‐covalently bound flavin adenine dinucleotide cofactor (FAD), and it contains a redox‐active cysteine motif (CAAC in human ALR). ALR forms homodimers that are held together by non‐covalent interactions and two disulfide bonds [81, 88].

In its active form, both CXXC motifs of ALR are oxidized. In a first step, the shuttle arm of ALR non‐covalently binds to the hydrophobic groove in MIA40 that normally serves as substrate binding site (“substrate mimicry”) (Fig. 5B) [81, 85]. This allows positioning of the oxidized CXXC motif of ALR close to the reduced CPC motif of MIA40. Then, C55 of reduced MIA40 attacks the oxidized CXXC motif in the ALR shuttle domain. A thiol‐disulfide exchange leaves MIA40 oxidized, and the shuttle CXXC motif reduced. The binding of both substrates and ALR to the same site in MIA40 appears to be independent of the MIA40 redox state as overexpression of ALR in cells suppresses substrate oxidation defining a “goldilocks” concentration of ALR in the IMS [46].

To reoxidize the shuttle CXXC‐motif, a further thiol‐disulfide exchange is required. To this end, the shuttle arm reaches over to the second subunit of ALR and shuttles electrons in an inter‐subunit transfer reaction onto the core CXXC of this subunit (Fig. 5B) [81]. The core CXXC motif is positioned closely to the FAD cofactor allowing its reoxidation. The flavin cofactor is thereafter present in its reduced form, FADH_2_. To reoxidize FADH_2_, electrons can be transferred to oxidized cytochrome *c*, and from there into complex IV of the respiratory chain (Fig. 5C) [89, 90, 91, 92].

The electron transfer between ALR and cytochrome *c* follows a very rapid collision‐type interaction that is sustained by a large surface area on ALR without the need for a canonical protein complex formation [90]. While during thiol‐disulfide exchange reactions, always two electrons are transferred, the shuttling of electrons from FADH_2_ to the heme cofactor of cytochrome *c* only involves transport of one electron. Different ideas have been put forward how this switch in transported electrons is facilitated. One idea is the stepwise transport from FADH_2_ onto two cytochrome *c* molecules resulting in the temporary existence of a FAD radical. Another idea stems from the comparatively short distance of the FAD cofactors in the two subunits of the ALR homodimer. It has been proposed that the two electrons from one FADH_2_ might distribute among them allowing the concomitant rapid transfer of single electrons onto two molecules cytochrome *c* [93].

Alternatively, FADH_2_ can also be reoxidized directly by molecular oxygen resulting in the generation of H_2_O_2_ as a by‐product (Fig. 5D). This reaction is up to a 100‐fold slower than the transfer of electrons onto cytochrome *c* but might become important upon accumulation of reduced cytochrome *c* [89, 90, 91]. In yeast, electron transfer has also been described under anoxic conditions. In this case, electrons are transferred onto the soluble fumarate reductase Osm1 (Fig. 5D) [94]. This metabolic electron acceptor uses the electrons from ALR to reduce fumarate to succinate. Such a backup pathway under low oxygen conditions has not been described for the human disulfide relay system, and it might also never have to work under truly anoxic conditions, but in principle other enzymes such as peroxiredoxins that have been detected in the IMS [95] or the glutathione pool might act as electron acceptors. If such anoxic electron acceptors exist in human cells, they might become important in hypoxic stem cell niches or during cancer development.

### Quality control in oxidation‐dependent IMS protein import

**Fig. 6:**
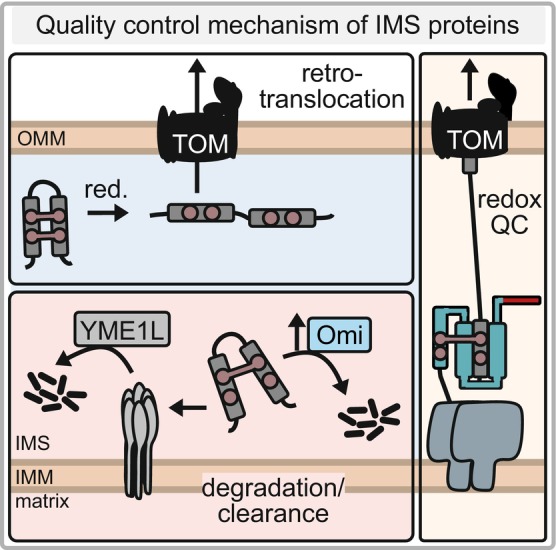
Quality control of disulfide relay substrates. IMS protein unfolding and accumulation leads to the activation of chaperones and proteases. This can trigger estrogen receptor activity and upregulates the IMS protease Omi/HtrA2. The i‐AAA protease YME1L is a main player for clearance of unfolded or misfolded IMS proteins. In yeast, it has been shown that IMS proteins can be reduced and retro‐translocate back to the cytosol for proteasomal degradation. MIA40‐substrate intermediates can be resolved by reducing factors or C53 of MIA40 while the substrate is partially translocated through the OMM. This results in substrate backsliding to the cytosol, proteasomal degradation and thereby prevents TOM pore clogging and accumulation of blocked permanently substrate‐engaged MIA40.

The mechanism of disulfide relay‐mediated protein import also opens the opportunity for quality control steps. First, the long dwelling time of precursors in the cytosol goes along with the proteasomal degradation of a significant share of the precursors in the cytosol, which has been reported in particular for the yeast system (Fig. 6) [70, 71]. In human cells, the N‐terminal processing by DPP8 and 9 sensitizes precursors to proteasomal degradation, which competes with the import into mitochondria [50]. Currently, it remains unclear to which extent, under which conditions, and for which proteins *en route* degradation takes place in human cells.

Second, a redox quality control step during translocation across the OMM has been reported. The formation of a mixed disulfide bond between MIA40 and its substrates while they are still translocating through the TOM channel offers a time‐window in which resolution of this disulfide bond would still allow to retrotranslocate, extract and proteasomally degrade the precursor (Fig. 6) [37]. This disulfide bond resolution can be driven, for example, by reducing machineries in the IMS, including thioredoxins and glutaredoxins that can reduce the mixed disulfide bond. It has indeed been shown that the amounts of these enzymes need to be tightly balanced as their overexpression can also prevent productive disulfide‐dependent import [37, 96]. This quality control step prevents the accumulation of blocked permanently substrate‐engaged MIA40 that cannot contribute to IMS protein import anymore.

Another layer of proteasomal surveillance of the IMS proteome includes the reduction of mature oxidized proteins, and their retrotranslocation from the IMS to the cytosol where they undergo proteasomal degradation (Fig. 6) [97]. This process has been reported to occur in yeast upon shifting cells from a carbon source necessitating respiration (glycerol) to a carbon source that allows fermentation and in yeast also leads to the suppression of expression of mitochondrial genes (glucose). In yeast, this process might well‐complement the Yme1‐dependent control of the mature IMS proteome that has been proposed earlier [98, 99]. In human cells, IMS proteome control by YME1L likely also impacts disulfide relay substrates. Moreover, in human cells, induction of the protease Omi/HtrA2 and the proteasome has been reported to take place under conditions of folding stress in the IMS [100]. Both help to degrade IMS proteins including disulfide relay substrates.

### Human diseases associated with the mitochondrial disulfide relay

Mutations in disulfide relay substrates are associated with human disease. We present here only a small selection of the associated pathologies, where we feel that the underlying molecular mechanisms are best understood.

NDUFB10 is an accessory structural subunit of complex I of the respiratory chain [101]. It is located in the P_D‐a_ module of the complex, and important for the association of this module with the neighboring P_P‐b_ module. Deletion in cell culture leads to a loss of complex I function [102, 103]. In a patient, compound heterozygous mutations of NDUFB10 (one mutation led to a premature stop codon and absent protein, the second mutation replaced cysteine 107 with a serine residue) resulted in lowered complex I activity in muscle, heart, and liver, and fatal infantile lactic acidosis and cardiomyopathy [104]. C107 is part of one of two disulfide bonds in the mature protein, and its mutation impaired oxidation and efficient mitochondrial accumulation of NDUFB10, and resulted in degradation of non‐imported precursors. However, the impaired protein levels were not observed in all tissues to the same extent; liver and in particular fibroblasts were affected to a lesser extent (even having normal complex I activity) [104]. This is interesting as it indicates that oxidation‐dependent import might still be possible with the remaining disulfide bond of NDUFB10 but is so slow that in most cells, quality control systems remove the protein before successful incorporation into complex I.

Another protein involved in complex I assembly is NDUFAF8 [105, 106]. It has been found to interact in the mitochondrial matrix with NDUFAF5 [106, 107]. The NDUFAF5–NDUFAF8 complex hydroxylates the complex I structural subunit NDUFS7 at an early stage in the assembly of complex I [108]. Loss of NDUFAF8 in cell culture caused a dramatic reduction in NDUFAF5 protein levels and complex I activity [106, 107]. Clinically, three unrelated patients were identified who harbored homozygous or compound heterozygous mutations in the NDUFAF8 gene leading to Leigh syndrome and complex I deficiency [105]. Interestingly, NDUFAF8, although a substrate of the disulfide relay, is found in the mitochondrial matrix. It is representative of a class of disulfide relay substrates (including also CHCHD1, CHCHD2, and CHCHD10) that follow a two‐step import pathway linking IMS and matrix import systems [106]. Weak targeting sequences drive their TIM23‐dependent matrix import, and *en route*, allow exposure of the precursors to the disulfide relay, which oxidizes the proteins.

Different mutations of TIMM8A are associated with Mohr–Tranebjaerg syndrome or deafness‐dystonia syndrome [109, 110, 111, 112, 113, 114]. TIMM8A belongs to the class of small TIMM proteins. It forms a heterohexameric complex with its partner TIMM13, and functions in importing and shuttling hydrophobic proteins into the IMM via the TIM22 pathway [115, 116]. The syndrome can be associated with sensorineural deafness, dystonia, loss of vision, and behavioral disturbances. One of the reported mutations led to an exchange of cysteine residue 66 by a tryptophan (C66W). This cysteine is involved in forming one of two disulfide bonds in mature TIMM8A. The mutation did not prevent IMS import of the protein indicating again that formation of one disulfide bond might be sufficient to import at least small amounts of protein into the IMS. The mutation did, however, impair the subsequent assembly of TIMM8A with its partner TIMM13 [117]. Moreover, the protein was destabilized and rapidly degraded.

Mutations in the disulfide relay machinery have also been reported as disease‐causing. For example, different AIFM1 mutations lead to combined oxidative phosphorylation deficiency resulting in a severe mitochondrial encephalomyopathy and the Cowchock Syndrome [118, 119, 120, 121]. Recent data indicate that most of these phenotypes of AIFM1 mutations might be due to the resulting absence of MIA40, which depends on AIFM1 for its mitochondrial import [25]. At least in cell culture, the loss of AIFM1 could be fully complemented by the overexpression of MIA40 [25, 27, 122].

An arginine to histidine (R194H) mutation in ALR resulted in autosomal recessive mitochondrial myopathy with cataract and combined respiratory chain deficiency and developmental delay [123, 124]. Subsequent structural and biochemical analyses of ALR then revealed that this arginine is located at the dimer interface of ALR and involved in multiple stabilizing hydrogen bonds. Consequently, the R194H mutation affected protein stability and flavin binding; interestingly, it did not impact enzyme activity of ALR [125, 126].

## Open questions

Our understanding of the human disulfide relay has grown tremendously over the past 15 years. Still, many open questions remain. For one, many of the (patho)physiological links and functions of the disulfide relay like its involvement in the hypoxia response [127], its role in mitochondrial fission [128, 129] or cytosolic iron–sulfur cluster assembly [130, 131] are not easy to explain with our mechanistic knowledge of the system. They might also well be associated with the different proposed extramitochondrial functions in particular of ALR [132, 133].

Second, our understanding of cytosolic processes in mitochondrial disulfide relay‐dependent import is still very poor, and in particular the targeting but also the handling of precursors in the cytosol during unperturbed and stressed conditions awaits further characterization. Some hints came from elegant yeast work that demonstrated that a stress response, the “unfolded protein response activated by mistargeting of proteins” (UPRam) can be triggered by the cytosolic accumulation of disulfide relay substrates. This then resulted in a decrease in global translation rates and increased proteasome activity [134]. In human cells, such responses have not been assessed in detail.

Lastly, the interaction between AIFM1 and MIA40 might link metabolism and disulfide relay‐dependent protein import. AIFM1 requires the binding of NADH or NADPH for dimerization and only the dimer can import and bind MIA40 [25, 27]. Shifts in the ratio of NAD(P) towards the oxidized forms might thereby suppress dimerization and slow down MIA40 import and attenuate disulfide relay functionality. It will be exciting to explore this link further.

## Conflict of interest

The authors declare no conflict of interest.

## Author contributions

JR and CZ wrote the text and designed the figures.
